# Platelet-rich fibrin (PRF) gel modified by a carbodiimide crosslinker for tissue regeneration

**DOI:** 10.1039/d2ra00985d

**Published:** 2022-05-05

**Authors:** Fatemeh Karimi, Esmaeil Biazar, Saeed Heidari-Keshel, Bahareh Pourjabbar, Mohammad Reza Khataminezhad, Shervin Shirinbakhsh, S. Yasaman Zolfaghari-Moghaddam

**Affiliations:** Tissue Engineering Group, Department of Biomedical Engineering, Islamic Azad University Tonekabon Branch Tonekabon Iran kia_esm@yahoo.com +00981154271105; Department of Tissue Engineering and Applied Cell Sciences, School of Advanced Technologies in Medicine, Shahid Beheshti University of Medical Sciences Tehran Iran; Department of Biology, Islamic Azad University Tonekabon Branch Tonekabon Iran; Department of Biomedical Engineering, Science and Research Branch, Islamic Azad University Tehran Iran

## Abstract

Platelet-rich fibrin (PRF) as a rich source of effective growth factors has been used as a scaffold in tissue regeneration. It is known that PRF exhibits rapid degradability against enzymes, which should be decreased using crosslinking agents to reduce the release rate of growth factors and increase the effectiveness of tissue regeneration. In this study, a carbodiimide crosslinker with different concentrations (0.01%, 0.05%, 1%, and 2%) was used to modify and improve the properties of PRF gel. The crosslinked gels were evaluated with analyses such as SEM, swelling, degradability, mechanical strength, release test, cytotoxicity, and cell adhesion. The results showed that with increasing crosslinker concentration, the morphology of the fiber structure changes drastically, the swelling rate decreases from 300% (control) to 160% for the crosslinked gel, the degradation time for the control sample increases from 8 days to more than two weeks for the crosslinked gel, and the Young's modulus increases from 0.15 MPa (control) to 0.61 MPa for the crosslinked samples. Growth factors also showed lower release with increasing crosslinking ratio. Cytotoxicity assays demonstrated that by increasing the crosslinker concentration to 1% w/v, no cytotoxicity was observed. Cellular studies with DAPI staining showed that the cells penetrated well into the gels and were well distributed, especially in gels with lower crosslinker concentrations. In addition, the modified PRF gel can be used as a scaffold for tissue regeneration.

## Abstract 2

1-Ethyl-3-(3-dimethyl aminopropyl) carbodiimide hydrochloride (EDC) as a crosslinker can improve the physical and mechanical properties of PRF gel by forming covalent bonds.

## Graphical abstract

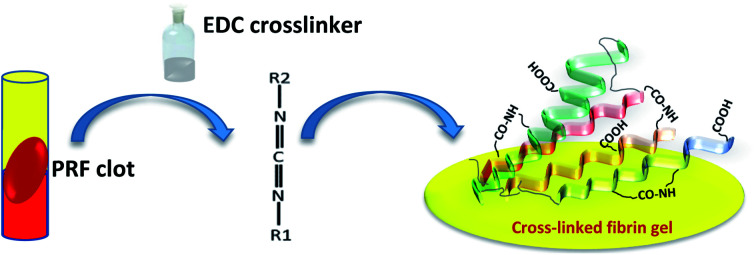

## 1. Introduction

The development of biomaterials and the use of new methods to improve, repair and regenerate soft and hard tissues are the main goals of tissue engineering.^1^ Growth factors, as one of the pillars of tissue engineering, have an important role in the process of tissue repair and regeneration.^2^ Platelet-rich fibrin (PRF), as an autologous biomaterial rich in growth factors with simplified processing without biochemical blood handling, is increasingly being studied and used by specialists to repair and regenerate tissues.^3,4^ PRF was first developed in France by Choukroun *et al.*^5^ for specific use in oral and maxillofacial surgery. This technique requires neither anticoagulants nor bovine thrombin (or any other gelling agent). It is nothing more than centrifuged blood without any addition, which makes it possible to avoid all the restrictions posed by French law related to blood-derived product implantation. PRF gels containing platelet-specific and non-platelet-specific proteins as a membrane or scaffold have exhibited slow release of growth factors, such as the vascular endothelial growth factor (VEGF), transforming growth factor (TGF), and platelet-derived growth factor (PDGF), for at least 7 days *in vitro*.^6,7^ Increasing the time of effectiveness of such factors can help the process of tissue regeneration.^7,8^ Further, crosslinking the fibrin fibers with different crosslinkers can prolong the degradability time of the PRF membrane and increase its enzymatic resistance, provided that it does not disrupt the biological activity of the growth factors so that it can have a positive effect on recovery time. Methods such as heat compression have been able to reduce the surface area and porosity of the PRF membrane and delay its degradation by up to 4 weeks.^9^

Charanthanyakun *et al.*^10^ showed a reduction in the degradation time of PRF membranes by the heat compression method. The degradation time increased from 10 days for the control sample to 17 and 16 days for temperatures of 90 and 100 °C, respectively. Kawase *et al.* reported the same results by the heat treatment of PRF membranes at 90 °C. The results showed a doubling in the time of degradation compared to that of the control samples, while the treated samples in the animal model showed a degradation rate of approximately 4 weeks.^8^ Yu *et al.* showed different results by examining the biological and mechanical properties of heat-treated PRF membranes. They used two heating processes, namely single-side heating and double-side heating at 90 °C for 10 seconds using a metal plate heater. Compared to the control, the heated samples showed a significant reduction in size and weight due to the denser microstructure. During the double-sided heating of the membranes, not only were the mechanical properties and degradation improved but the cell viability and proliferation were also reduced.^11^ Studies have also reported that certain crosslinking agents, such as genipin, can improve heterograft tissues such as bovine pericardial tissues^9^ and tendon cells^12^ as well as biomaterials such as chitosan,^13^ fibrin-agarose hydrogel (FAH),^14^ and PRF.^15^ Sulistiawati *et al.* showed that the degree of enzymatic degradation of the crosslinked PRF membrane with genipin (1%) was significantly lower than that of the control sample, and the samples treated for 72 hours showed the lowest degree of degradation. Genipin can be resistant to fibrinolytic degradation due to the formation of covalent bonds with free primary amines.^4^ Heterobifunctional carbodiimides, and specifically 1-ethyl-3-(3-dimethyl aminopropyl) carbodiimide hydrochloride (EDC), have been used as chemical crosslinkers for the improvement of biomaterials.^15^ EDC is a zero-length crosslinker as it does not become incorporated into the macromolecule, thus reducing the likelihood of cytotoxic effects.^16,17^ The aim of this study was to evaluate the possibility of using EDC with different concentrations as a crosslinking agent for the fixation of PRF used in regenerative therapy, which is represented by different analyses.

## 2. Materials and methods

### 2.1. Preparation of the PRF clot

**Fig. 1:**
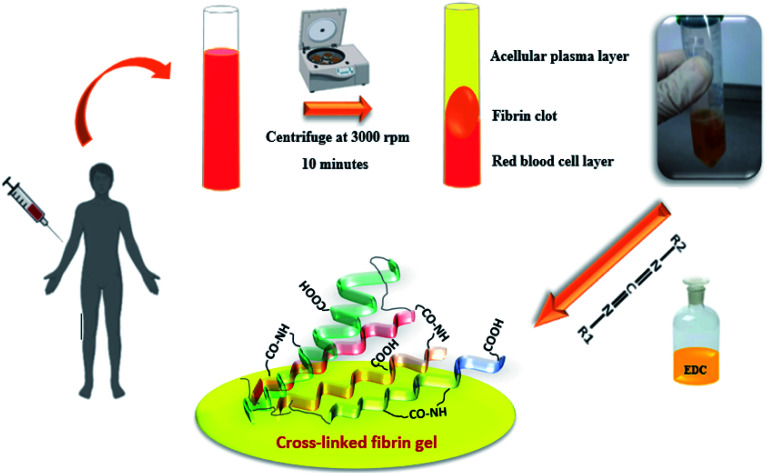
Schematic of the formation process of the PRF clot and crosslinking.

Blood samples were collected from healthy male volunteers (27 to 67 years old) using 21-gauge needles equipped with a conventional vacuum plain glass tube. PRF was prepared according to Dohan *et al.*^3^ Briefly, 10 mL blood samples were drawn into a BD Vacutainer without an anticoagulant, and the samples were then centrifuged at 3000 rpm for 10 min. This procedure separates the blood samples into different fractions, such as a red blood cell (RBC) base at the bottom, a fibrin clot (PRF) in the middle, and an acellular plasma (platelet-poor plasma [PPP]) supernatant layer at the top. The PRF clot was preserved at −18 °C. The prepared fibrin gels containing growth factors were modified with the EDC crosslinker at different concentrations (0.01, 0.05, 1 and 2% w/v). The gels were immersed into an EDC solution (ethanol/water: 90/10) for 24 hours under neutral conditions (pH: 7), then washed several times to remove all unreacted materials. All processes are shown in Fig. 1.

### 2.2. FTIR analysis

The un-crosslinked and crosslinked gels were weighed and mixed by grinding with potassium bromide (KBr). The mixture was pressed into tablets to prepare for measurement using Fourier transform infrared spectroscopy (FTIR) (BoMem Model MB100) at room temperature (25 °C) and a scan range of 500–4000 cm^−1^.

### 2.3. Morphological studies

Changes in the microstructure and morphology of the un- and crosslinked samples were analyzed by scanning electron microscopy (SEM) (ESEM Philips XL30). The samples and control (without crosslinking) were cut into small pieces and then covered with a thin layer of gold. The imaging process was performed at 20 V.

### 2.4. Physical and mechanical studies

#### 2.4.1. Tensile strength

To evaluate the mechanical properties of the gel, we performed a uniaxial tensile test. Accordingly, the samples with thicknesses of 3 mm were cut into 3 mm × 10 mm strips and blocked onto a tensile tester (SANTAM Co, Iran) with clamps in a 50 N load cell. The dimensions of the sample were measured using digital calipers and recorded in the tensile testing software. The load cell was calibrated, and the sample was pulled with an extension rate of 5 mm min^−1^ until the sample broke. This test was repeated three times.

#### 2.4.2. Swelling and degradability tests

For the swelling test, the samples were weighed when dry (*W*_d_) and then immersed in PBS for 7 days. The samples were taken out of the PBS at different times and individually weighed (*W*_w_). The surface water was gently blotted using filter paper before weighing the immersed samples. The swelling ratio was obtained using the following equation (eqn (1)), and the samples were analyzed in triplicate:

1 Swelling ratio (%) = (*W*_w_ − *W*_d_)/*W*_d_ × 100%

For the degradability test, the samples with dimensions 1 cm × 1 cm and weight 0.25 g were accurately weighed and placed in trypsin (0.05% w/v) and EDTA (1% w/v) solutions and incubated at 37 °C. The samples were weighed and the trypsin solution was refreshed every 24 hours. The difference in weight represents enzymatic degradation. The weight loss percentage was calculated according to the following formula (eqn (2)):

2 Weight loss% = (*W*_i_ −*W*_f_)/*W*_i_ × 100

where *W*_i_ is the initial dry weight of skin (mg) and *W*_f_ is the dry weight of the recovery fragment of skin after degradation (mg). The percentage of weight loss was obtained from the average of three experiments.

#### 2.4.3. Release test

Four pieces of PRF (3 mm diameter) with different concentrations of the EDC crosslinker (0, 0.01, 0.05, and 1% w/v) were incubated at 37 °C in a 48-well plate with 1 mL PBS per well. The scaffolds were incubated at 37 °C in a 48-well plate with 1 mL PBS per well. At specific intervals (24, 48, 96, and 168 hours), 50 μL aliquots were taken out and kept at −20 °C. Simultaneously, 50 μL PBS was added to fix the final volume at 1 mL. A micro BCA assay was used to determine the amount of protein in each sample. A microplate reader was used to measure the optical density at 570 nm (BioTek, USA). The absorbance values of the samples were normalized using the fibrin control as a reference. The data were then converted to μg mL^−1^ using a calibration curve created with BSA as a standard.

### 2.5. Cell studies

#### 2.5.1. Cytotoxicity assay

Regarding previous methods,^18^ the proliferation of cells was analyzed by the measurement of living cell numbers using the MTT assay. The C619 (skin BALB/c mouse-derived; NCBI Code: C619) epithelial cell line (Iranian Pasteur Institute cell bank) was used to assess cell viability and proliferation rate. In brief, the cells were plated in 96-well culture plates at a density of 1 × 10^4^ cells per well. Circular samples with 3 mm diameter were cut using a punch under sterile conditions. After 24, 48, and 72 hours cell incubation, 3-(4,5-dimethylthiazol-2-yl)-2,5-diphenyl-2*H*-tetrazolium bromide (MTT) (5 mg mL; Sigma Chemical Co, USA) was added to each well of the monolayer cultures. The dishes containing cultured cells were then incubated in a humidified atmosphere containing 5% CO_2_ at 37 °C. Finally, formazan crystals were dissolved using 100 μL DMSO overnight and the optical density was measured at 570 nm using a microplate reader (Rayto Life and Analytical Sciences Co., Shenzhen, China).

#### 2.5.2. Immunostaining analysis

For the *in vitro* detection and tracking of cells in the gel, epithelial cells were labeled with the nuclear stain 4′,6-diamidino-2-phenylindole dihydrochloride (DAPI; Sigma-Aldrich).^19^ Briefly, DAPI was added to the culture medium for 2 hours when the cells were 80% confluent. Then, the cells were harvested using trypsinization and prepared for seeding on the gels for 72 hours. The specimens were cut and fixed in formalin 10%. DAPI fluorescence was analyzed using a fluorescence microscope (Nikon Instruments, USA).

### 2.6. Statistical analysis

The values for all samples were evaluated as the mean ± standard deviation (SD). Statistically significant differences, *i.e. p* < 0.05 in a single time group and *p* < 0.01 between groups, were investigated using the Student's *t*-test and one-way analysis of variance (ANOVA) with Tukey's post hoc multiple comparison test for all groups.

## 3. Results and discussion

**Fig. 2:**
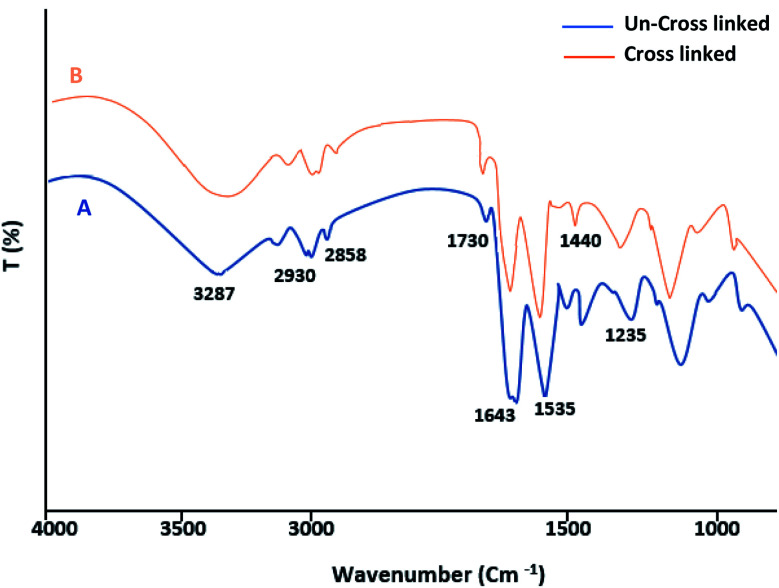
FTIR spectroscopy of un-crosslinked (A) and crosslinked (EDC 1%) fibrin gel (B).

The FT-IR spectrum of the fibrin clots exhibited a very similar profile to the characteristic bands of protein structures. The C=O stretching vibration of the polypeptide backbone results in amide I and II bands with a characteristic absorption peak in the range of 1643 and 1535 cm^−1^. The absorption peak of the amide III band is a combined peak generated by the C–N stretching vibration and in-phase N–H bending vibration and appears within the range of 1235 cm^−1^ (Fig. 2A). After the EDC crosslinking (Fig. 2B), the wavenumbers of the fibrin bands are almost the same as those of the un-crosslinked samples. This may suggest that the secondary structure of fibrin was not affected. Although no significant increment in the N–H band was observed (amide A band), the decrement in the amide I intensities suggests a more covalent crosslinked structure and a weakened carboxyl group related to the formation of new bonds.

**Fig. 3:**
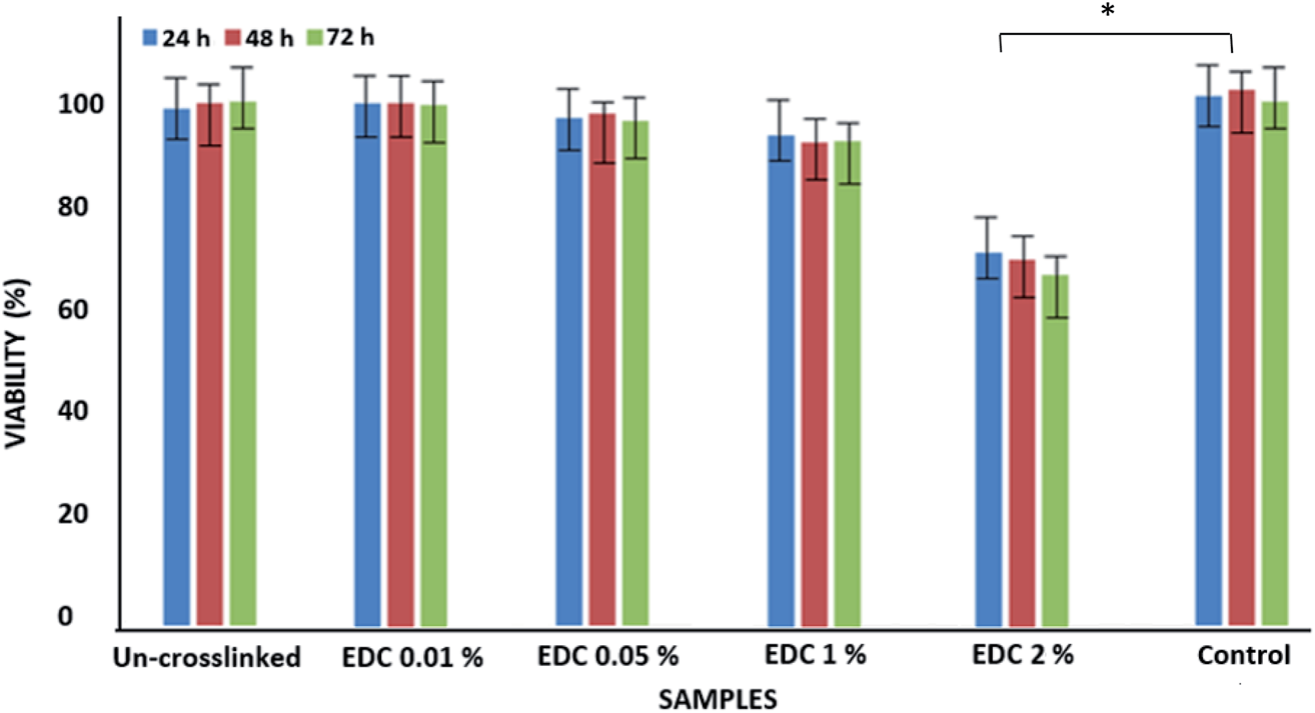
Viability of epithelial cells on un-crosslinked and crosslinked gels with different EDC concentrations. *p < 0.01.

Fig. 3 shows the results from the MTT test obtained from the un-crosslinked and crosslinked samples. Cell viability after 72 hours did not show a significant difference between the un-crosslinked and crosslinked samples at all concentrations except for the crosslinked samples with a concentration of 2% EDC compared to the control samples (TCPS) (*p* < 0.01). Cytotoxicity has been shown to depend on crosslinker concentration. Our results show that increasing the EDC amount to 1% w/v does not increase toxicity. Gamboa-Martínez *et al.*^20^ demonstrated that direct exposure to GP in the cells can promote their death in high concentrations. At 7 days of culture, cells reached confluence in all the gels, confirming the feasibility of GP as a crosslinker for fibrin.

**Fig. 4:**
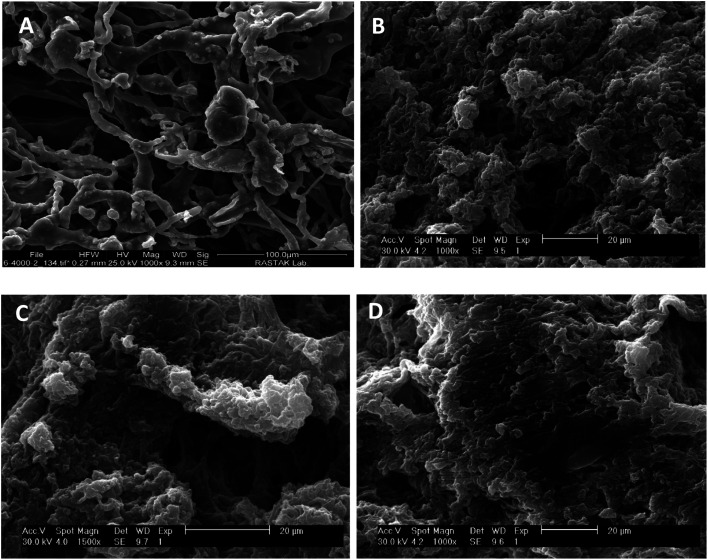
SEM images of the morphology of the un-crosslinked PRF gel (A) and crosslinked PRF gel at different concentrations: 0.01% w/v (B), 0.05% w/v (C), and 1% w/v (D).

Fig. 4 shows images of the samples modified with different crosslinking degrees using SEM analysis. The results show that the un-crosslinked sample has a regular fibrillar morphology with a porous structure (Fig. 4A). However, the internal structures of the samples were altered after crosslinking with the different concentrations of the EDC crosslinker (Fig. 4B–D). The samples modified with low concentrations of the crosslinker (0.01 and 0.05% w/v) show compressed fibers, but for the samples crosslinked with a high EDC ratio (1% w/v), the fibers are completely linked and compacted such that no porosity is seen between the fibers. Other studies have also shown changes in the morphology of the fibers with crosslinkers. Gamboa-Martínez *et al.*^20^ crosslinked fibrin gels with genipin (GP) as a natural crosslinker. The un-crosslinked fibrin showed a homogeneous open porous fibrillar structure with a fibril diameter around 300 nm. After crosslinking, the morphology of the gels changed, and increase in the amount of GP reduced the porosity of the samples and produced an agglomeration of fibers, especially with the highest GP concentration (5 mM).

**Fig. 5:**
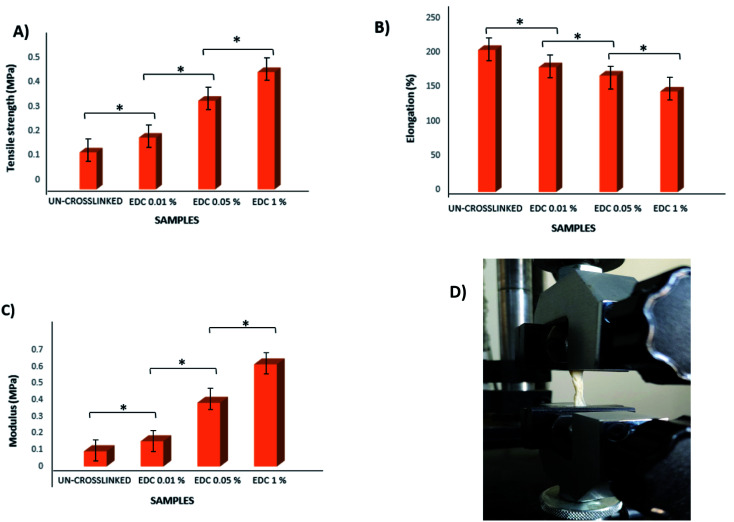
Diagrams of the tensile strength, elongation, and modulus of the un-crosslinked and crosslinked gels with different concentrations (A, B, and C) and simulated tension of the clot using dynamic mechanical analysis (D). *p < 0.01.

According to the obtained results, the tensile strength of all samples increased with increasing crosslinker concentration. The maximum tensile stress for the un-crosslinked gel was 0.15 MPa (with Young's modulus = 0.09 MPa), whereas the maximum tensile stress for the crosslinked samples with the highest crosslinker concentration at 1% w/v was 0.477 MPa (with Young's modulus = 0.61 MPa) (Fig. 5). All samples showed significant differences from each other (*p* < 0.01). Of course, slight differences in the values obtained from our results and other studies may be due to differences in parameters such as the thickness of the samples as well as the composition of the ECM.

**Fig. 6:**
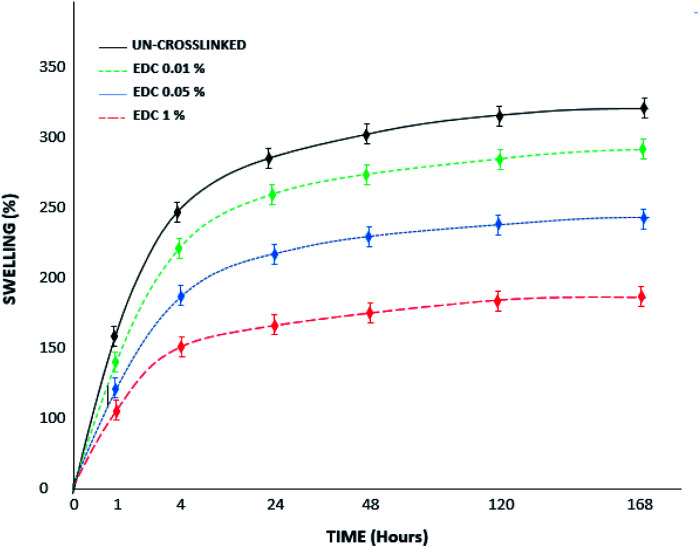
Swelling percent of the un-crosslinked and crosslinked gels with different concentrations over 7 days.

Fig. 6 shows the swelling behavior of the samples before and after the crosslinking process at different concentrations. The swelling ratio of all samples increased as time passed and almost achieved stability on day 5. The swelling ratios were calculated to be 300% and 160% for the un-crosslinked and crosslinked gels at the highest concentration of 1% w/v, respectively. The results show that swelling decreases with increasing crosslinker concentration, which can be due to intramolecular and intermolecular reactions of the functional groups in the chains, which ultimately cause more compression of the chains and reduced water penetration. The water absorption capacity of fibrin hydrogels crosslinked with genipin in the study of Gamboa-Martínez *et al.* was lower than that of pure fibrin.^20^ Based on the SEM images with both genipin^20^ and EDC (in our study), the porosity of the systems and the water sorption capacity of the samples decreased with increasing crosslinker concentration, while the mechanical strength increased according to previous research.^21^

**Fig. 7:**
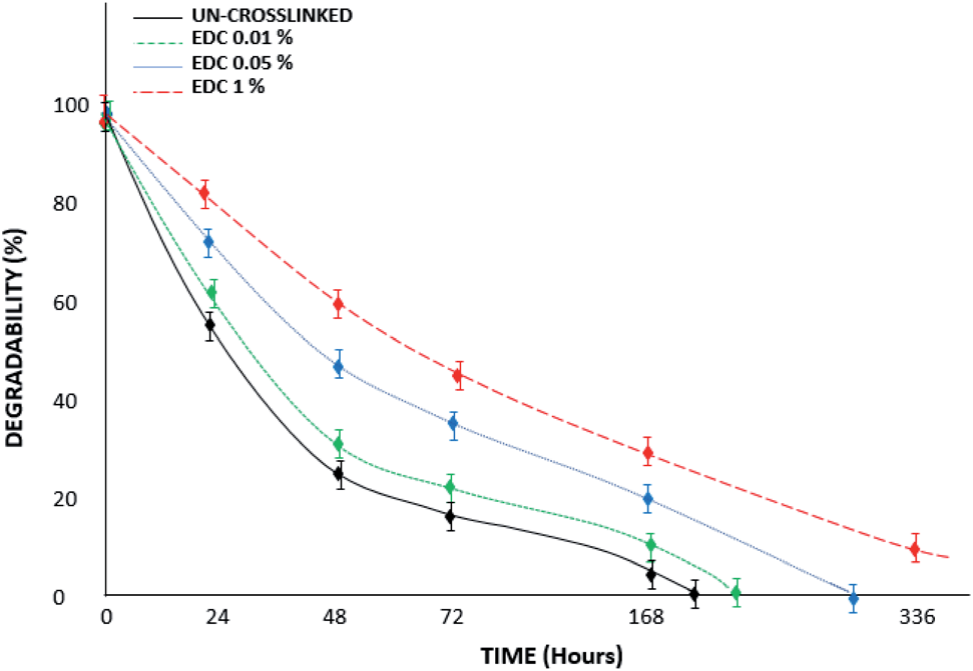
Degradability percent of the un-crosslinked and crosslinked gels with different EDC concentrations in trypsin/EDTA media over 14 days.

Fig. 7 shows a diagram of the degradation of the samples in collagenase media over 14 days of study. The results proved that the un-crosslinked samples had been destroyed on day 8; however, the degradation rate of the gels decreased with increasing crosslinker amount. According to the results, the modified samples with high concentrations (1% w/v) of crosslinker show less weight loss, such that they remain stable for up to 14 days and do not disappear completely. In the study by Sulistiawati *et al.*,^4^ the enzymatic degradation degree of PRF crosslinked with genipin was significantly lower than that of un-crosslinked PRF. It was found that chemical crosslinking with genipin for 72 hours had the lowest degradation degree. Genipin-crosslinked fibrin clots are resistant to fibrinolytic degradation,^22^ similar to our results with the EDC crosslinker. EDC molecules react with free primary amines and carboxylic acids through a series of reactions to form covalent crosslinks. EDC-based covalent crosslinks are resistant to enzymatic degradation and reduce effective molecular weight between crosslinks.

**Fig. 8:**
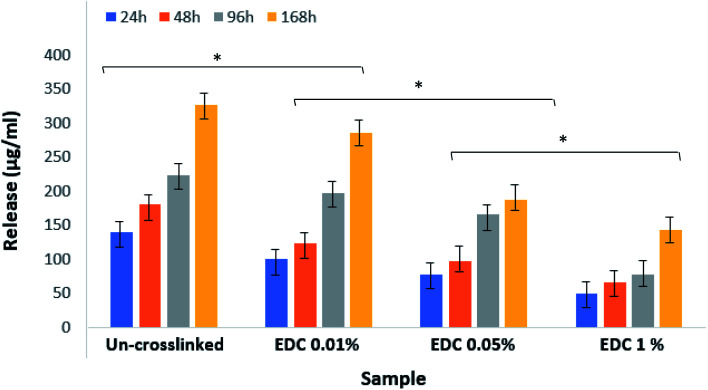
Release amount of growth factors from un-crosslinked and crosslinked gels with different EDC concentrations. *p < 0.01.

Fig. 8 shows the results related to the release of growth factors in PBS. The samples were not degraded over 7 days under these conditions, and therefore everything in the medium is related to the growth factors released from the fibrin gel. The figure shows a significant difference between the groups (*p < 0.01) as well as within groups at different times (*p* < 0.05). The diagrams show that the amount of release decreases with increasing crosslinker concentration.

**Fig. 9:**
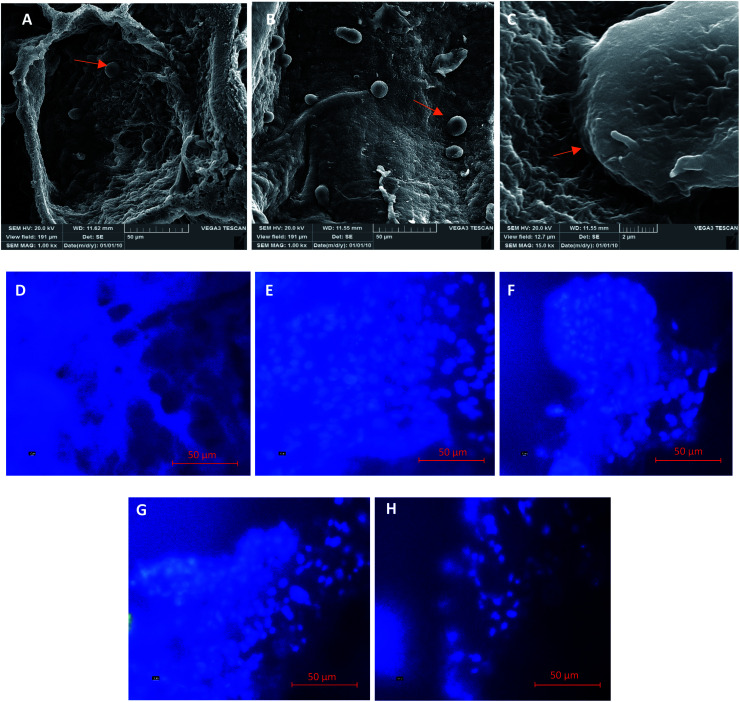
SEM images of platelets and epithelial cells on the crosslinked sample with EDC 0.05% w/v at different magnifications (A, B, and C). DAPI images of the un-crosslinked samples without (D) and with epithelial cells (E) and the crosslinked samples with EDC 0.01% (F); EDC 0.05% (G); and EDC 1% (H). Mag. 50 μm.

Fig. 9A–C show microscopic images of platelets and the presence of epithelial cells on the samples. The images show that a few epithelial cells adhere on the gel surface due to the gel structure of fibrin and the penetration of the cell into it. Therefore, for further cellular investigation, the SEM images were ignored and we performed intra gel studies by DAPI staining. Fig. 9D is related to the cell-free gel, and Fig. 9E–H are ascribed to the un-crosslinked and crosslinked gels with 0.01, 0.05, and 1% w/v EDC crosslinker, respectively. The DAPI-stained nucleus of epithelial cells and their accumulations are well shown in the figures. The results demonstrate that the adhesion and presence of epithelial cells inside the gels decreased with increasing crosslinker concentration. This could be due to the crosslinking of the peptides within the chain, the participation of functional groups, and finally the disappearance of most of the effective functional groups (amines and carboxylic acids) in the fibrin gel. Additionally, the high strength of the crosslinked samples prevents the cells from penetrating into the gel.

## 4. Conclusion

The EDC chemical crosslinker with a desirable concentration can stabilize tissue structures and improve their physical and mechanical performance. In this study, different concentrations of the EDC crosslinker were used to modify some of the properties of PRF gel, which can be used as a biological scaffold. The results showed that increasing the crosslinker concentration improves mechanical strength, swelling, and degradation time; conversely, it decreases the morphology of the matrices and the coherence and compactness of the layers and fibers. Significant differences were observed in the cytotoxicity of the gels with increasing crosslinker concentration. The cell distribution within the gel also decreased with increasing crosslinker concentration. Crosslinked gels can be used in treatments that do not need long-term stability but must be durable in the early phase, such as in socket preservation, closure of oroantral communication, soft tissue wound dressing and tissue regeneration.

## Conflicts of interest

The authors state no conflict of interest and have received no payment in the preparation of this manuscript.

## Supplementary Material
